# Advanced Deep Learning Analysis of Cognitive, Clinical, and Bio‐Psychosocial Markers for Rapid Dementia Risk Detection to Inform Prevention trials in Africa

**DOI:** 10.1002/alz70860_100052

**Published:** 2025-12-23

**Authors:** Chinedu T Udeh‐Momoh, Investigators of The Africa‐FINGERS Program

**Affiliations:** ^1^ FINGERS Brain Health Institute, Solna, Stockholm, Sweden, Brain and Mind Institute, Aga Khan University, Nairobi, Kenya, Division of Public Health Sciences, Wake Forest University, School of Medicine, Winston‐Salem, NC, USA

## Abstract

**Background:**

With global dementia cases projected to triple by 2050, over **60% occurring in low‐ and middle‐income countries (LMICs)**; there is an urgent need for **contextually relevant dementia risk prediction models** in African populations. The **Aga Khan University Brain and Mind Institute (AKU‐BMI) are collaborating with global stakeholders and have launched several** pioneering regional initiatives that integrate deep phenotyping of **cognitive, clinical, and bio‐psychosocial markers** to enhance early detection and risk stratification for **Alzheimer's disease and related dementias (ADRD)** in African older adults. These initiatives focus on developing and validating culturally sensitive diagnostic tools while creating a **pipeline for rapid recruitment into the Africa‐FINGERS program, the first dementia risk reduction trial in Africa**.

**Methods:**

The projects employ **advanced diagnostic capabilities**, including neuroimaging (**MRI, FDG‐PET**), **fluid biomarkers** (blood and saliva), and **culturally adapted neuropsychological assessments**, to deeply phenotype participants across the dementia continuum. Cardiometabolic, vascular, and lifestyle risk factors (often underdiagnosed yet critical in African populations) are a focal point. Additionally, pilot **ethnographic research has identified unique contextual risk factors** that are rarely explored in global dementia studies, such as **malnutrition, household hunger, lack of spirituality, multidimensional poverty and social isolation**, which may significantly impact cognitive aging and well‐being in African settings.

**Result:**

Preliminary analyses reveal strong associations between **elevated systolic blood pressure, fasting glucose, stroke history, smoking, and low social engagement** with increased dementia risk. Moreover, a **significant discrepancy** between self‐reported and objectively measured cardiometabolic conditions suggests **widespread underdiagnosis**, underscoring the **urgent need for targeted prevention and intervention strategies** in African populations.

**Conclusion:**

The ongoing e**pidemiological cohort studies at AKU‐BMI are critical for the rapid detection and identification of at‐risk individuals** for recruitment into **Africa‐FINGERS**, a landmark clinical trial for dementia prevention in Africa. Our innovative model identifies **unique, locally relevant dementia risk factors** and aligns them with **global diagnostic frameworks**, **setting a precedent for LMIC‐adapted dementia research**. This **scalable, data‐driven approach** not only strengthens **precision prevention efforts in Africa** but also provides **a replicable framework for global dementia prevention initiatives, particularly for underserved and diaspora populations**.

